# Transcriptome Analysis of Silkworm, *Bombyx mori*, during Early Response to *Beauveria bassiana* Challenges

**DOI:** 10.1371/journal.pone.0091189

**Published:** 2014-03-11

**Authors:** Chengxiang Hou, Guangxing Qin, Ting Liu, Tao Geng, Kun Gao, Zhonghua Pan, Heying Qian, Xijie Guo

**Affiliations:** 1 Sericultural Research Institute, Jiangsu University of Science and Technology, Zhenjiang, Jiangsu, China; 2 Sericultural Research Institute, Chinese Academy of Agricultural Sciences, Zhenjiang, Jiangsu, China; Uppsala University, Sweden

## Abstract

Host–pathogen interactions are complex processes and it is a central challenge to reveal these interactions. Fungal infection of silkworm, *Bombyx mori*, may induce a variety of responsive reaction. However, little is known about the molecular mechanism of silkworm immune response against the fungal infection. To obtain an overview of the interaction between silkworm and an entomopathogenic fungus *Beauveria bassiana*, Digital Gene Expression profiling, a tag based high-throughput transcriptome sequencing method, was employed to screen and identify differentially expressed genes (DEGs, FDR≤0.001, ∣log_2_ratio∣≥1) of silkworm larvae during early response against *B. bassiana* infection. Total 1430 DEGs including 960 up-regulated and 470 down-regulated ones were identified, of which 627 DEGs can be classified into GO categories by Gene Ontology (GO) analysis. KEGG pathways analysis of these DEGs suggested that many biological processes, such as defense and response, signal transduction, phagocytosis, regulation of gene expression, RNA splicing, biosynthesis and metabolism, protein transport etc. were involved in the interaction between the silkworm and *B*. *bassiana*. A number of differentially expressed fungal genes were also identified by mapping the sequencing tags to *B. bassiana* genome. These results provided new insights to the molecular mechanism of silkworm immune response to *B. bassiana* infection.

## Introduction

The silkworm, *Bombyx mori,* is a typical lepidopteran insect and has important economical value in many developing countries. It has also contributed enormously to the study of insect genetics and immunology [1], [2], [3]. *Beauveria bassiana* is a major pathogenic fungus for silkworm. It usually infects silkworm by penetrating the cuticle, then causes white muscardine disease to the silkworm and enormous damages to the sericultural industry.

The process of fungi infecting insects can be divided into three stages: surface adhesion, cuticle penetration, replication in vivo, leading to the death of host [4]. *B. bassiana* spores adhering to the silkworm cuticle usually germinate and invade into silkworm in 6–8 h and the larvae will die about 2–3 days later. In the third stages of *B. bassiana* infection, oily spots appear on the body of the silkworm larvae, which is the typical symptom of white muscardine. Entomopathogenic fungi generally infect insects by direct penetration of the cuticle, followed by their multiplication in the hemocoel [5], [6]. They can be recognized and fought by the innate immune system of hosts. Insect response to fungi infection has attracted extensive attention because fungi have been widely used in biological control of pests [7]. The interactive and responsive mechanisms of silkworm against *B. bassiana* are still poorly understood, which is an obstacle to the development of new control measures against this destructive disease of the silkworm.

Silkworm is a good model for insect immune response due to its easy rearing and operation for experiments. Hou *et al* (2011) found that 77 differentially expressed genes participated in the procedure of infection of *B. Beauveria* by suppression subtractive hybridization (SSH) methods, but their potential roles in molecular mechanism of infection and innate immunity of insects were still unknown [8]. Digital Gene Expression (DGE) profiling [9], a transcriptome analytic technology, which can screen and identify millions of short RNAs and differentially expressed genes (DEGs) in a sample without prior annotations, has been employed in many domains [10], [11]. To gain an overall view of the transcriptome profiling during the silkworm response against *B. bassiana* infection, we employed the Illumina Genome Analyzer platform to perform transcriptome analysis, then systematically analyzed the gene expressional profiles in the infected and control silkworm larvae, including the up- and down-regulated DEGs, Gene Ontology (GO) categories and KEGG Pathways. The results are reported as part of our effort in exploring the molecular mechanism of insect immune response against *B. bassiana* infection.

## Results

### Confirmation of Infection

At approximately 36 hours post-inoculation (hpi), oily spots appeared on the body of the larvae, which is a typical symptom of the muscardine. The infected larvae all died in 60 hpi. The disease was further confirmed by the observation of the short hypha in hemolymph under microscope and appearance of white powdery conidia on the dead larvae body.

### Digital Gene Expression (DGE) Profiles

**Figure 1:**
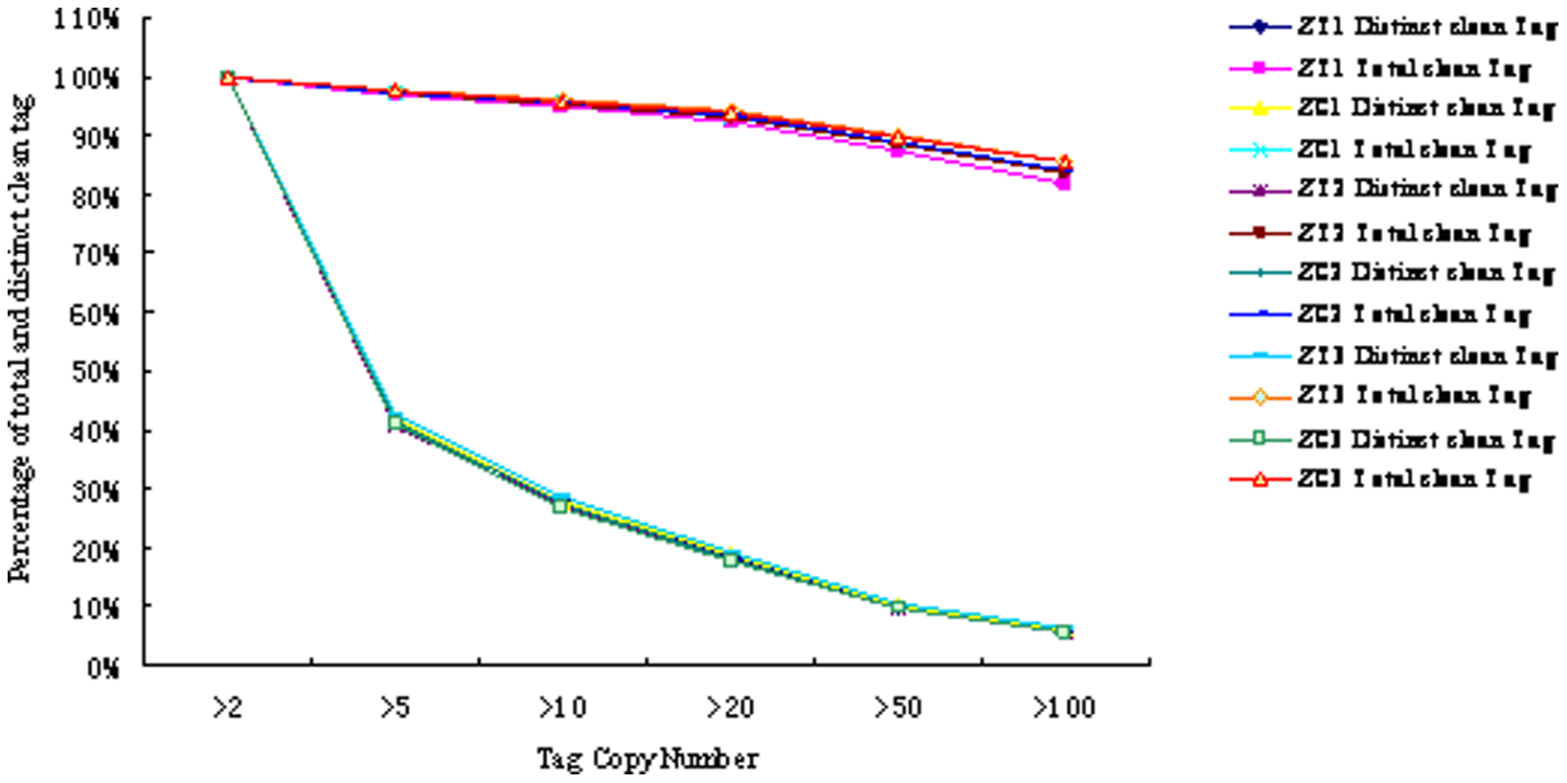
Abundance and categories distribution of the total and distinct clean tags of each library.

In order to obtain a global view of transcriptome related with early response of the silkworm to *B. bassiana* infection, we constructed six DGE tag libraries, numbered correspondingly as ZT1/ZC1, ZT2/ZC2 and ZT3/ZC3 (ZT was the tested samples and ZC was the control ones), from the six total RNA samples isolated from the infected and control silkworm larvae at 8, 15 and 24 hpi by high-throughput sequencing with Solexa Sequencing Chip (flowcell). 3.9∼4.9 million raw tags were generated for different library and 94.98%∼96.89% (over 3.7 million) of them in each library were clean tags. Approximately 0.07 million of the raw tags in each library were distinct tags. The statistics of the DEG tags is shown in Table 1. In this analysis, the total number of clean tags is the sum of all the clean tags and the number of distinct clean tags is the number of different clean tags. The abundance and categories of the total and distinct clean tags showed similar tendencies between the different libraries (Fig 1). In each library, 5–7% of the distinct tags were tags with copy numbers more than 100, 33–38% of the distinct tags with copy numbers between 5 and 50 and 57–60% of the distinct tags with copy numbers from 2 to 5. These results reflect the principle that a small number of mRNAs are expressed at a very high abundance, while the majorities are expressed at a very low level [12], which indicated that our DGE dataset was normally distributed.

**Table 1:** Distribution of tags and genes in the DGE libraries.

Summary	Distribution of Tags and Genes	8h		15h		24h	
		ZC1	ZT1	ZC2	ZT2	ZC3	ZT3
Raw Data	Total	4819322	3969200	4610798	4413692	4970777	4506968
	Distinct Tags	192503	181983	191012	159421	193560	169209
Clean Tags	Total number	4588715	3794277	4379323	4276434	4720369	4331281
	Distinct Tag number	75276	71217	71504	74016	72933	63977
All Tags Mapping to Genes	Total number	1338847	1197135	1139740	1413597	1163957	1069771
	Total % of clean tags	29.18%	31.55%	26.03%	33.06%	24.66%	24.70%
	Distinct Tag number	14204	14427	13413	15433	13054	12282
	Distinct Tag % of clean tags	18.87%	20.26%	18.76%	20.85%	17.90%	19.20%
Unambiguous Tags Mapping to Gene	Total number	1215887	1076803	1026438	1209093	1033172	935814
	Total % of clean tags	26.50%	28.38%	23.44%	28.27%	21.89%	21.61%
	Distinct Tag number	13709	13932	12937	14890	12510	11727
	Distinct Tag % of clean tags	18.21%	19.56%	18.09%	20.12%	17.15%	18.33%
All Tag-mapped Genes	number	5615	5715	5507	6026	5242	4964
	% of ref genes	34.39%	35.00%	33.73%	36.90%	32.10%	30.40%
Unambiguous Tag-mapped Genes	number	5348	5444	5205	5726	4903	4687
	% of ref genes	32.75%	33.34%	31.88%	35.07%	30.03%	28.70%
	Total number	5473		5576		4847	
Mapping to Genome	Total number	2610102	2026647	2633663	2321337	2866928	2564040
	Total % of clean tags	56.88%	53.41%	60.14%	54.28%	60.74%	59.20%
	Distinct Tag number	39858	38169	39256	39814	37464	35838
	Distinct Tag % of clean tags	52.95%	53.60%	54.90%	53.79%	51.37%	56.02%
Unknown Tags	Total number	639766	570495	605920	541500	689484	697470
	Total % of clean tags	13.94%	15.04%	13.84%	12.66%	14.61%	16.10%
	Distinct Tag number	21214	18621	18835	18769	22415	15857
	Distinct Tag % of clean tags	28.18%	26.15%	26.34%	25.36%	30.73%	24.79%

### Analysis of Tag Mapping

It is a very important step to annotate sequences and reveal the molecular events behind the gene expression for the tags matched to genes [13]. For tag mapping, a reference tag database containing 55055 reference tags,54394 unambiguous reference tags and 13328 genes with CATG site was constructed from silkworm database (ftp://silkdb.org). All the raw data of sequence has been submitted to the SRA website (http://www.ncbi.nlm.nih.gov/Traces/sra/) with an accession number of SRX376906. All the clean tags were aligned to the reference tag database, 17–20% of the distinct tags in all the six libraries were uniquely mapped to the genes and 21–28% of existing transcripts were matched by tags. Total 428923 distinct tags were obtained from the six DGE libraries, of which 82813 were mapped to genes (Table 1). The unambiguous tags mapped to the reference tag database generated 5473, 5576 and 4847 tag-mapped genes for the 8, 15 and 24 hpi libraries respectively. 53–60% of the clean tags were mapped to silkworm genome. While 12–16% of the clean tags could not be mapped to the reference tag database and they were designated as the unknown tags.

Since Solexa Sequencing can distinguish transcripts originated from DNA double-strands and employing the strand-specific nature of the sequencing tags obtained, we found that 86–88% of genes have sense transcripts and 53–56% of genes have antisense transcripts (Table S1), and the genes with bidirectional transcripts ranged from 4687 to 5726 and those with antisense-stand specific transcripts ranged 2522 to 3239. By comparison, the ratio of sense to antisense strand transcripts was approximately 1.6∶1 for all the libraries. This suggested that the high number of antisense transcript were detected and the antisense transcriptional regulation of the *B. bassiana*- induced immune response in the silkworm were also strong.

We analyzed the sequencing saturation of each library to estimate whether the sequencing depth was sufficient for the transcripts coverage or not. Analysis showed that the number of genes mapped by unambiguous clean tags increased along with the number of genes mapped by all clean tags. When the total tag number reached up to approximately 2 M, the number of detected genes mapped by unambiguous clean tags was saturated (Fig S1).

### Analysis of Differentially Expressed Genes

**Figure 2:**
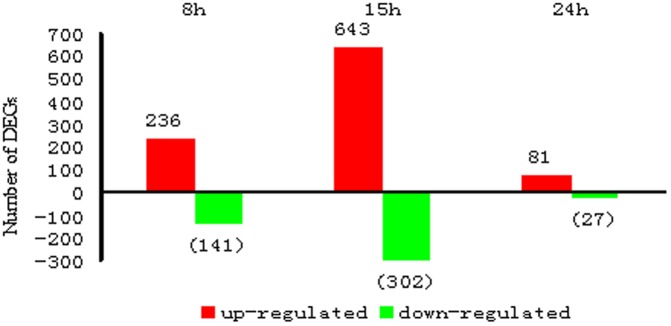
Differentially Expressed Genes in three time points.

Differentially expressed genes (DEGs) distinguished at the early stage of infection may provide an important clue to the host immune response against the *B. bassiana* infection. We firstly normalized the expression abundance of tag-mapped genes by counting the number of transcripts per million (TPM) clean tags, then used False Discovery Rate (FDR) ≤0.001 and |log_2_Ratio|≥1 as a threshold to identify differentially expressed genes during the infection course. Analysis showed that number of the identified DEGs differed in each time point of 8, 15 and 24 hpi (Fig 2). Total 1430 genes altered expression as compared to the control samples, of which, 960 genes were up-regulated and 470 genes were down-regulated.

**Figure 3:**
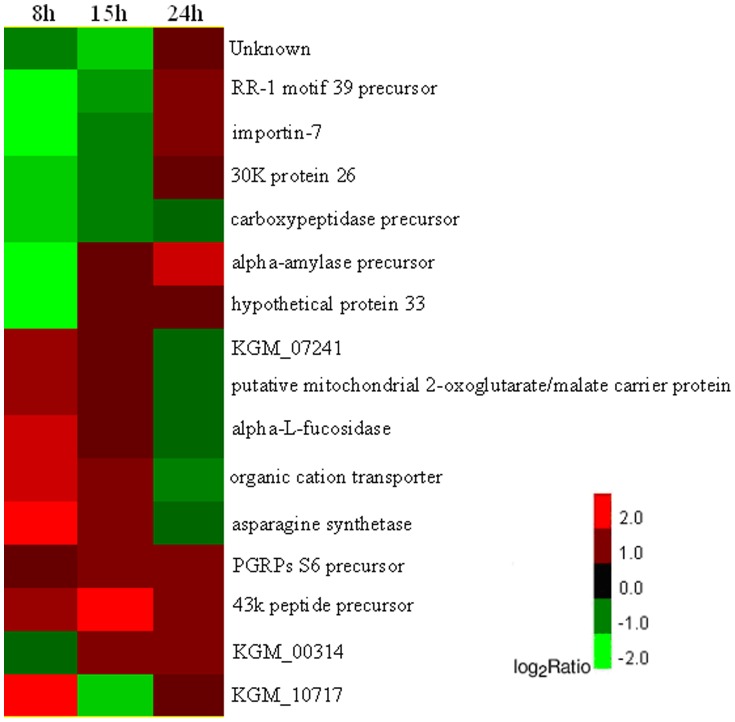
Clustering analysis of the intersection of DEGs in three time points. Each column represents a time point, each row represents a gene. Expressional differences are shown in different colors. Red means up-regulated and green means down-regulated.

**Figure 4:**
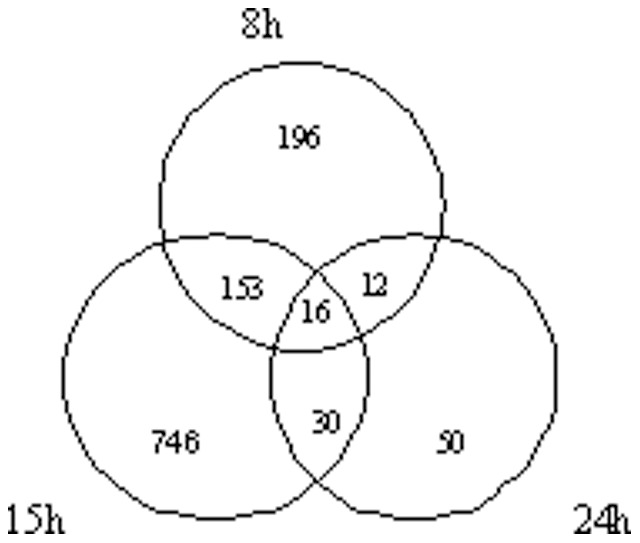
Number of common differentially expressed genes between different time points post-inoculation.

Clustering analysis of the DEGs among the time points revealed that 16 common unigenes showed differential expression at all the three time points of 8, 15 and 24 hpi (Fig 3), among which only one was down-regulated at all the three time points. 169 common unigenes showed differential expression at both the time points of 8 and 15 hpi, of which 103 DEGs were up-regulated, 29 down-regulated and the expressional regulation of the remaining 37 DEGs was different at the two time points, i.e. the expression of 11 DEGs was significantly suppressed at 8 hpi while more highly up-regulated at 15 hpi and the other 26 DEGs was opposite. 46 common unigenes showed differential expression at both the time point of 15 and 24 hpi, of which 8 DEGs were up-regulated, 4 down-regulated and the expressional regulation of the remaining 35 DEGs was different at the two time points, namely, the expression of 22 DEGs was more highly up-regulated at 15 hpi while significantly suppressed at 24 hpi and the other 12 DEGs was opposite. 28 common unigenes showed differential expression at both the time points of 8 and 24 hpi, of which 11 DEGs were up-regulated, 2 DEGs were down-regulated and the expressional regulation of 15 DEGs was different at the two time points, i.e. the expression of 10 DEGs was significantly suppressed at 15 hpi while more highly up-regulated at 24 hpi and the other 5 DEGs was opposite (Fig 4).

In these 731 DEGs with GO annotation, some can be classified into more than one GO annotation and some common DEGs were also identified at more than one time points. For example, 8 common DEGs were identified at all the three time points of 8, 15 and 24 hpi, only one of them was involved in cellular components, but 6 in molecular functions and 5 in biological processes respectively (Table S2). 84 common DEGs were identified at both the time points of 8 and 15 hpi, of which, 37 was classified into cellular components, 72 into molecular functions and 56 into biological processes respectively (Table S2). The 14 common DEGs identified at both the time points of 8 and 24 hpi include 3 ones with annotation of cellular components, 11 ones with molecular functions and 11 ones with biological processes, respectively (Table S4). Furthermore, 22 common DEGs were identified at both the time points of 15 and 24 hpi, including 9 with annotation of cellular components, 20 with molecular functions and 13 with biological processes, respectively (Table S3). Therefore, considering the common DEGs identified between different time points, the exact number of DEGs with GO annotation should be 627.

Some differentially expressed genes indentified in our previous study on the percutaneously infected silkworm were also indentified in the present experiment [8], [14], such as heat shock proteins, ribosomal proteins, transferrin, elongation factors, ATP synthase, genes encoding the related ubiquitin and cytochrome, lysozyme, cecropin-B precursor and so on. The newly identified genes in the present study included those encoding some kinds of hypothetical KGM proteins, hemolymph proteinase, lipase, importins, peptidoglycan recognition proteins, glucan recognition proteins, toll receptor, some kinds of serine proteases and their precursor, and so on. The genes that encode peptidoglycan recognition proteins and glucan recognition proteins were related with immune recognition of host, the genes that encode importins and other transporters were related with the transportation of nutrition, and the genes that encode lysozyme, cecropin-B precursor, hemolymph proteinase, serine proteases, ubiquitin and cytochrome were anti-microbial peptides or proteins with anti-microbial functions, whereas the genes that encode ribosomal proteins, elongation factors, and ATP synthase were involved in the pathologic process. Furthermore, ribosomal proteins and toll receptor participated in signal transductions.

### Fungal Related Factors Involved in Host-pathogen Interactions

To reveal the profiles of *B. bassiana* gene expression during infection, tags from DGE libraries were mapped to genome of *B. bassiana* (http://genome.jgi.doe.Gov/Beaba1/Beaba1.home.html). Because the tatol RNA was extracted from the silkworm larvae at the early stage of infection, the majority of RNA samples were from the silkworm and only a small portion of the DGE tags (from 0.05% to 0.68%) from three libraries were perfectly mapped to *B. bassiana* genes (Table S5–S7). This phenomenon indicated that those *B. bassiana* genes were expressed during its infection to the silkworm.

50, 71 and 60 *B. bassiana* genes were identified at 8, 15 and 24 hpi respectively. Comparing the expression levels of those genes among the three time points revealed that most fungal genes were up-regulated at 15 and 24 hpi (Table S5, S6), implying their vital roles in the process of the fungal infection. While, at 15 and 24 hpi, the number of up- and down-regulated genes was almost the same (Table S7).

### KEGG Pathways Influenced by *B. bassiana* Infection

The KEGG pathway analysis identified 10, 20, 18 pathways at the time points of 8, 15 and 24 hpi (P-value≤0.05), respectively (Table 2). Except Metabolic pathway, all other KEGG pathways differed at the three time points. However, five pathways, namely ribosome, aminoacyl-tRNA biosynthesis, spliceosome, proteasome, antigen processing and presentation were presented at 8 and 15 hpi, and glutathione metabolism is another common pathway at 15 and 24 hpi.

**Table 2:** KEGG Pathways identified at 8, 15 and 24

Pathways	P-value			Pathway ID
	8h	15h	24h	
Ribosome	3.98E-08	0.000143		ko03010
Aminoacyl-tRNA biosynthesis	7.74E-05	0.00195		ko00970
Spliceosome	0.000279	0.028973		ko03040
Tryptophan metabolism	0.005329			ko00380
Proteasome	0.007075	0.010694		ko03050
Metabolic pathways	0.017395	0.018192	0.001307	ko01100
Lipoic acid metabolism	0.017684			ko00785
Antigen processing and presentation	0.019309	0.003816		ko04612
Selenocompound metabolism	0.029117			ko00450
Amoebiasis	0.0306			ko05146
Epstein-Barr virus infection		0.00025		ko05169
Basal transcription factors		0.000765		ko03022
RNA transport		0.003447		ko03013
Protein processing in endoplasmic reticulum		0.006429		ko04141
Cytosolic DNA-sensing pathway		0.012407		ko04623
Nicotinate and nicotinamide metabolism		0.013031		ko00760
Glycosylphosphatidylinositol(GPI)-anchor biosynthesis		0.013075		ko00563
Folate biosynthesis		0.013075		ko00790
RNA polymerase		0.013144		ko03020
Pyrimidine metabolism		0.016574		ko00240
Glutathione metabolism		0.016831	0.001939	ko00480
Glycolysis / Gluconeogenesis		0.019966		ko00010
Ubiquinone and other terpenoid-quinone biosynthesis		0.034649		ko00130
Citrate cycle (TCA cycle)		0.046267		ko00020
Pancreatic secretion			3.5E-06	ko04972
Glycerolipid metabolism			0.002065	ko00561
Protein digestion and absorption			0.006188	ko04974
Glycine, serine and threonine metabolism			0.0071	ko00260
PPAR signaling pathway			0.00815	ko03320
Fat digestion and absorption			0.009042	ko04975
Riboflavin metabolism			0.010229	ko00740
Lysosome			0.015538	ko04142
Steroid biosynthesis			0.021532	ko00100
Prostate cancer			0.023792	ko05215
Cell adhesion molecules (CAMs)			0.027836	ko04514
Renin-angiotensin system			0.031335	ko04614
Bacterial invasion of epithelial cells			0.033143	ko05100
Hypertrophic cardiomyopathy (HCM)			0.037792	ko05410
Vitamin B6 metabolism			0.038064	ko00750
Alzheimer's disease			0.047765	ko05010

In these pathways, total 89 genes, including 44 up-regulated DEGs and 45 down-regulated ones were involved in 5 significantly enriched KEGG pathways (Qvalue≤0.05) (Table 3). They are pancreatic secretion, Epstein-Barr virus infection, ribosome (this pathway was identified at both 8 hpi and 15 hpi, respectively), spliceosome and aminoacyl-tRNA biosynthesis. Except Epstein-Barr virus infection and pancreatic secretion, most DEGs in other pathways were down-regulated.

**Table 3:** DEGs involved in the significantly enriched KEGG Pathways at 8, 15 and 24

	Pathway Name	Q-value	P-value	No. of genes	
				Up-regulation Down-regulation	
8h	Ribosome	6.80E-06	0.0000	2	12
	Aminoacyl-tRNA biosynthesis	6.62E-03	0.0001	4	5
	Spliceosome	1.59E-02	0.0003	6	9
15h	Ribosome	0.0286	0.0001	5	11
	Epstein-Barr virus infection	0.0286	0.0003	16	11
24h	Pancreatic secretion	0.0004	0.0000	11	1
	Total			44	45

### DEGs and Pathways Identified at 8 hpi

377 DEGs including 236 up-regulated and 141 down-regulated ones were identified at this time point (Fig 3). As shown in Table S2, 94 DEGs had GO component annotation, 158 DEGs had GO function annotation and 135 DEGs had GO process annotation. Among the DEGs with GO process annotation, expression levels of those related to metabolic process (alpha-L-fucosidase, cystathionine gamma-lyase), gene expression (SNF4/AMP-activated protein kinase gamma subunit, chymotrypsin inhibitor CI-8A), defense and response (moricin I, lysozyme precursor) were up-regulated. Some DEGs were down-regulated, such as those associated with respiration (dihydrolipoamide succinyltransferase component of 2-oxoglutarate dehydrogenase, NADH-ubiquinone oxidoreductase 39 kda subunit), mitosis (small nuclear ribonucleoprotein polypeptide, ribosomal protein L37) and so on.

In the ten pathways identified at this time point, amoebiasis, antigen processing and presentation pathways were up-regulated, which might regulate host defense. While expression levels of most identified DEGs were down-regulated in ribosome, aminoacyl-tRNA biosynthesis and spliceosome pathways, which may be affected by infection of *B. bassiana* (Table 3).

### DEGs and Pathways Identified at 15 hpi

At this time point, 945 DEGs were identified, of which 643 were up-regulated and 302 down-regulated (Fig 3). As shown in Table S3, 263 DEGs had GO component annotation, 411 DEGs had GO function annotation and 337 DEGs had GO process annotation, respectively. Among the DEGs with GO process annotation, expression level of some DEGs was down-regulated, including 8 DEGs in metabolic process, RNA splicing (LSM Sm-like protein family member). While some DEGs were up-regulated in protein transport (mitochondrial import inner membrane translocase), response to stimulus (RAD52 protein, heat shock protein hsp20.8), translation (eukaryotic initiation factor 4E-1, eukaryotic translation initiation factor 3 subunit I), peptidyl-arginine modification (putative protein arginine N- methyltransferase), phagocytosis (SLY-1 homologous, nonclathrin coat protein gamma1-COP), reproduction (putative voltage-dependent anion-selective channel isoform 1, cell cycle checkpoint kinase 2, kinesin-like protein Ncd), regulation of gene expression (transcription factor A, transcription initiation factor TFIID subunit 6, RNA-binding protein lark), signal transduction (phospholipase d, putative regulator of g protein signaling), and so on.

At this time point, pathways were related to metabolic, virus infection, antigen and genetic information processing. These pathways were aminoacyl-tRNA biosynthesis, RNA transport, Epstein-Barr virus infection, antigen processing and presentation, protein processing in endoplasmic reticulum, RNA polymerase, spliceosome and basal transcription factors, respectively. Compared with the time point of 8 hpi, only six common pathways, i.e. spliceosome, aminoacyl-tRNA biosynthesis, metabolic pathways, antigen processing and presentation, proteasome and ribosome were identified, while others were specific to the time point of 15 hpi.

### DEGs and Pathways Identified at 24 hpi

At this time point, 108 DEGs including 81 up-regulated and 27 down-regulated ones were identified (Fig 3). As shown in Table S4, 15 DEGs had GO component annotation, 39 DEGs had GO function annotation and 28 DEGs had GO process annotation, respectively. In the DEGs with GO process annotation, the expression levels of the DEGs related to defense response (lysozyme precursor, cecropin-B precursor, moricin I), RNA splicing (peptidyl-prolyl cis-trans isomerase h), metabolic process (P260, serine protease-like protein precursor), gene expression (eukaryotic translation initiation factor 3 subunit E) were up-regulated, while the expression levels of the DEGs related to biological regulation (importin-7) were down-regulated.

18 pathways were identified at this time point, most of them were up-regulated, such as lysosome related to immune response, PPAR signaling pathway related to inflammation inhibition, pancreatic secretion related to digestion and absorption of nutrition (protein, fat, vitamin and carbohydrate), amino acid (glycine, serine and threonine) metabolism. While less numbers of DEGs were involved in these pathways. Compared with the pathways at 8 and 15 hpi, only one common pathway was identified at all the three time pints, i.e. metabolic pathways.

### DEGs Related to Defense Response and Signaling Pathways

Some DEGs with immune and response function were identified at all the three time points, such as immune-related Hdd1, TNFSF13, moricin I, Cecropin B, lysozyme precursor and so on. Some DEGs participating in signaling pathways were also identified, such as MAPK signaling pathway (heat shock protein 70–14, putative protein phosphatase-5, TPR-repeat protein), Jak-STAT signaling pathway (PREDICTED: signal transducing adapter molecule 1-like, uncharacterized protein LOC100216501), Toll-like receptor signaling pathway (toll receptor, isochorismatase domain containing protein), p53 signaling pathway (hypothetical protein KGM_01522, putative f-spondin) and so on. Except peptidoglycan recognition protein S6 precursor, no other common DEGs with immune response were identified in these three time points, but several common DEGs namely moricin I, lysozyme precursor and β-1,3-glucan recognition protein 3 precursor were identified at both 8 and 24 hpi.

### Validation of DGEs by Real-time qPCR

**Figure 5:**
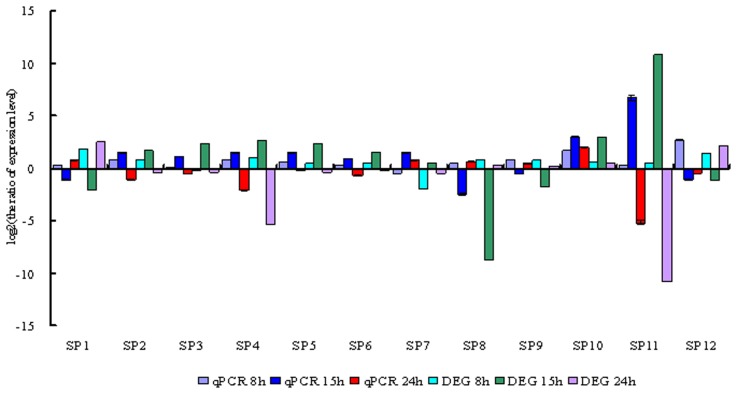
Comparation of Real-time qPCR detection with DGE profiling. SP1: neutral lipase. SP2: Bm8 interacting protein 2d-4 precursor. SP3: glucose transporter. SP4: hypothetical protein KGM_13211. SP5: troponin C. SP6: ecdysone-induced protein 63F 1. SP7: amidase. SP8: vacuolar ATP synthase subunit D. SP9: serine protease 13. SP10: putative protein phosphatase-5. SP11: hemolymph proteinase 18. SP12: peptidoglycan recognition protein S6 precursor.

Time course analysis of the gene expression was performed by real-time qPCR, to validate DEGs identified by Solexa Sequencing. Twenty two DEGs related with defense response and signal transduction and other functions were selected for the real-time qPCR detection, their transcript levels were compared and twelve of them were showed in Fig 5. Results showed that Real-time qPCR data of these genes were consistent with the DGE analysis. For example, both DGE and Real-time qPCR analysis showed the expression of the genes encoding cytochrome P450 CYP4G48, hemolymph proteinase 18, Bm8 interacting protein 2d-4 precursor, hypothetical protein KGM_13211, troponin C, ecdysone-induced protein 63F 1, asparagine synthetase were up-regulated in the *B bassiana* infected larvae at 8 and 15 hpi, while down-regulated at 24 hpi, especially the gene encoding hemolymph proteinase 18 was significantly more highly expressed at 15 hpi while more significantly suppressed at 24 hpi. Likewise, expression of the genes encoding glutathione S-transferase omega 1, heat shock protein hsp 19.9, neutral lipase, vacuolar ATP synthase subunit D, serine protease 13 and peptidoglycan recognition protein S6 precursor were up-regulated at 8 hpi while down-regulated at 15 hpi; the gene encoding serine protease precursor and putative protein phosphatase-5 were highly expressed at all the three time points; the gene encoding 30 kP protease A (43 k peptide) precursor was suppressed at 8 hpi but highly expressed at 15 and 24 hpi.

## Discussion

Although the silkworm systematic immunity responding to bacterial infection is extensively studied, little is known about the interaction of silkworm against fungal infection [8], [15]. In the insect immune system, except the known anti-fungal peptides and related recognition proteins [16], some other molecules which have anti-fungal activities may be involved. However, relevant research remains little, especially in the silkworm.

During the spores of *B. bassiana* infect silkworm, a complicated interactional process between host and pathogen may be involved, namely the host quickly recognizes the invaded pathogen and initiates immune response, while the pathogen must undergo complex survive attacks and multiply themselves in the host hemolymph. Several platforms for genome-wide screening have been used to study interactions between the host and the pathogen. Microarrays and SSH (suppression subtractive hybridization) are the most widely used methods. Many genes involved in pathogen recognition, signal transduction, immuno-response have been identified by microarray and SSH [8], [17], [18]. While, Digital Gene Expression Profiling can more economically and quickly capture the gene expression profiles of the whole genome of a certain tissue or a species under specific conditions.

In the present study, DGE Profiling was employed to identify differentially expressed genes (DEGs) involved in the interaction between silkworm and pathogenic fungus *B. bassiana*. The gene expression profiles were interpreted by Solexa Sequencing Chip (flowcell). By data processing, 5473, 5575 and 4846 unambiguous tag-mapped genes were generated at 8, 15 and 24 hpi respectively, from which 377, 945 and 108 DEGs were distinguished respectively. KEGG analysis identified 48 pathways and 6 significantly enriched pathways in these DEGs. Meanwhile, the ratio of the transcripts of sense to anti-sense strand was approximately 1.6∶1 for all the libraries. Sequencing tags mapped to the complementary strand of a gene suggest that its antisense strand also has transcripts and this gene may use the sense-antisense regulation and thereby the antisense transcripts may play an important role in gene expression and regulation. The DEGs identified against the fungal infection might be functional and play important roles in immune progress. Therefore, the present research focused on the DEGs of the silkworm because we think that they may not only be responsive to *B. bassiana* infection but also affect fungi proliferation in the host.

The analytic results of the significantly enriched KEGG pathways indicated that many genes involved in the pathways of pancreatic secretion and Epstein-Barr virus infection were up-regulated. Pancreatic secretion is related with the digestion and absorption of proteins, carbohydrates, fats and vitamins. The DEGs identified in the Epstein-Barr virus infection pathways include three Konjac glucomannans )KGMs), two Proteasomes and four Heat shock proteins (Hsps). KGM is a kind of neutral polysaccharides with excellent biocompatibility and biodegradable activities, which can form protective film, arrest absorption of glucide to lower blood cholesterol and sugar level, help with weight loss, promote intestinal activity and immune function [19]. Proteasome is a principal mechanism for regulating the specific protein synthesis and deleting wrong folded proteins. It is related with many cellular functions, such as cell cycle and division, differentiation and development, signal transduction, apoptosis, immune response and so on [20], [21]. Some lepidopteron larvae resist virus infection by selective apoptosis and sloughing off the infected cells [22], [23]. Hsps are molecular chaperones, essential for maintaining cellular functions and responding to a range of stress-related stimuli [24], [25]. Hsps may be potent activators of innate immune system, for they can stimulate production of proinflammatory cytokines mediated by CD14/TLR complex signal transduction pathways [26], [27]. Some of them have anti-apoptotic properties and can be therapeutic targets [28]. Some may be a part of the cellular innate antiviral immune responses [29] and increase immuno-regulation by activation of anti-inflammatory T-cells and natural killer (NK) cells, bind to Toll-like receptors (TLRs) on antigen-presenting cells and detect immune modulatory components in food as a readout [30]. Extra-cellular Hsps act as danger signals alerting the immune system to initiate an appropriate response [31], [32].

In our DGE study, the up-regulated DEGs including KGM, proteasome and Hsps were detected at 15 hpi, which suggests that after the invasion of *B. bassiana*, the host may reduce nutritional intake and promote immune function by the up-regulated KGM, activate immune cells by Hsp and TLRs to initiate defense and arrest proliferation of the fungus. Thus, the fungus must escape from the recognition and defense of silkworm and absorb and utilize the nutrition of the host for their multiplication.

We also found that the expression level of some genes involved in the pathways of ribosome, aminoacyl-tRNA biosynthesis and spliceosome, such as leucyl-tRNA synthetase, serine/threonine-protein kinase, glutamine, cysteine, arginine, ribosomal protein L13A and so on, were up-regulated at 8 hpi. In the process of immunological stress, amino acids are redistributed to tissues involved in immune response. They are used for the synthesis of inflammatory and immune proteins to support the proliferation of immune cell and other important components with defense functions. Then normal biological processes were disturbed by stimulation of the immune system, the requirements of specific amino acid can be induced [33].

Peptidoglycan recognition proteins (PGRPs) are one of the pattern recognition receptors (PRRs) which are much conserved and can recognize pathogen associated molecular patterns (PAMPs). PGRP-S6 is a kind of low molecular extracellular protein with short transcripts. PRRs activate prophenoloxidase (proPO) in the immune system of insect and poisonous melanin was translated into microorganism [34]. The up-regulated expression of PGRP-S6 precursor in the infected silkworm at 8 hpi implies that it functions in recognizing and binding to peptidoglycan then activating the host immune system.

Serine proteases (SPs) are proteolytic enzymes with important roles in innate immunity of arthropods, such as blood clotting, activation of proPO leading to melanin synthesis [35], [36] and proteolytic activation of Toll signaling pathway to induce production of antimicrobial peptides/proteins [37], [38]. SPs are normally inactive zymogens and sequentially activated once they recognized the invading microbial PAMPs. They become inactivated again once their functions were accomplished. Hemolymph proteinases (HPs) are one kind of serine proteinases. HP18 was expressed in hemocyte and its mRNA levels were increased at 24 h after a bacterial challenge, implying that it rapidly mediate defense responses upon microbial infection [39]. Similarly, the up-regulation of SP precursor and HP18 in the infected larvae may activate the immune system of the host against the invading fungus.

Importin 7, one of the multiple alternative functional receptors, belongs to the importin β family which mediate proteins into the nucleus [40], [41]. It also acts as chaperones to effectively suppress the aggregation of its basic import cargoes and its expression can be a key mediator of cell differentiation [42]. The up-regulated Importin 7 in the infected silkworm larvae may involve in importing some related proteins into the nucleus and mediating cell differentiation of host.

Cytochrome P450 s (mixed function oxidase) are a ubiquitous and complex superfamily of heme-containing enzymes that participate in metabolism of both endogenous and exogenous substrates [43]. Insect P450 s have several typical motifs of high sequence conservation, namely heme-binding region, helix-C, helix-I, helix-K and PERF [44]. There are 84 P450 s-like genes and 78 of them are functional genes in the silkworm. Most P450 s of silkworm are tandem arranged on chromosomes, and their expression level were up-regulated in the fat body after exposure to insecticides [45]. In our study, we found the relative expression level of P450 was up-regulated at 8 and 15 hpi but down-regulated at 24 hpi in infected larva, which implied that P450 also response to infection and proliferation of *B. bassiana*.

30 kP protease A (43 kD peptide), belongs to the elastase-like serine proteases, can selectively hydrolyze the 30 kD yolk protein of silkworm between Ser6 and Ala7 [46]. Its mRNA reached a maximum level at larval hatching and remained a low concentration during post-embryonic development [47]. In insects, 30 kD proteins belonging to lipoprotein of the vitellogenin family are involved in releasing diglyceride from the fat body, transporting lipid, sterols and hormones and providing nutritions for activities of eclosion and mating, and may play a role in defense against fungal infection [48]. In this study, the relative expression level of the 43 kD peptide precursor was down-regulated at 8 hpi but rapidly up-regulated at 15 hpi and remained a higher level afterwards in the infected larva, implying that it may hydrolyze 30 kD proteins to defend the silkworm against fungal proliferation.

Lysozyme is another kind of hydrolytic enzymes. It has catalytic activity cleaving the β-(1,4)-glycosidic bond of the bacterial peptidoglycan. The cleaved fragments can function as signal molecules and initiate immune response as they can be captured and transmitted by PGRPs in insect [49]. It was also involved in some other functions, such as mediating melanization of foreign targets [50], as a digestive enzyme using bacteria as a food source, scavenging cell debris by other antibacterial proteins functioning on bacteria [51]. The up-regulation of lysozyme in the present study implied that it may melanize or digest the invading fungus in the host hemolymph.

Bm8 interacting protein 2d-4 is one of the silkworm factors interacting with Bm8, a protein of an early gene and probably involved in viral DNA replication and/or transcription. At the early stage of BmNPV infection, Bm8 interacting protein 2d-4 was slightly up-regulated, then markedly decreased. It may be a receptor/ligand for cell signaling pathways or endocytosis. The interaction between these two proteins may be involved in entry and budding of virus particles. In this study, the relative expression level of the Bm8 interacting protein 2d-4 precursor was detected to be up-regulated at 8 and 15 hpi, but markedly decreased in the infected larvae at 24 hpi, implying that it may act as a signaling receptor and related with reproduction of fungal hypha.

Compared with the control, expression level of many genes was changed in the test group after *B. bassiana* infection. Similar phenomenon was also identified in the silkworm infected by other pathogens. Zhou *et al*. reported that SP precursor transcript level was notably higher in the susceptible silkworm strain from 0 to 72 hpi relative to nucleopolyhedrovirus infection by microarray [52], while Bao *et al*. showed that it was higher only at 12 hpi in the susceptible strain as compared to the resistant strain by SSH [53]. Our data showed that SP precursor transcript level was suppressed at 15 hpi, then up-regulated at 24 h pi. The roles of SP precursor in immune were not completely consistent with the results reported previously. Sagisaka *et al*. confirmed that the expression of Hsp significantly decreased after BmNPV infection [54], while Bao *et al* and Wu *et al*. found that Hsp transcript level was increased after BmNPV [53] and BmCPV [14] infection. The transcript levels of Hsps in our test were also up-regulated following the *B. bassiana* infection. These suggested that the same genes in the silkworm may have different responses against infection by different pathogens. Therefore, the functions of a gene in response to a specific pathogen infection required to be studied in details.

Although PCR-based cDNA screening and microarrays can identify a set of DEGs in plants and insects, much more DEGs can be identified by DGE system [10], [55], [56]. Since the results of gene expression analysis using different methods are not always consistent [57], the genes identified from DGE system also need to be validated by real-time qPCR.

In summary, with Digital Gene Expression profiling approach and analysis of differential gene expression, 1430 differentially expressed genes (DEG) were identified in the silkworm in the earlier stage of infection by *B. bassiana*. The function of some DEGs involved in immune and defense was discussed. The expression levels of putative lysozyme, PGRP-S6, SPs, HP18, P450 and Imp-7 were up-regulated at 8 hpi. It can be suggested that the up-regulated lysozyme hydrolyzed the invaded pathogen into fragments, the fragments were then be recognized by the recognition proteins PGRP-S6, SPs etc, and with the signal transduction by up-regulated Bm8 interacting protein 2d-4 precursor and glucose transporter etc, the immune system of the silkworm was activated. The proteins or their hydrolysate with immune function, such as SPs, HP18, P450, putative lysozyme and 30 kP protease A precursor etc may function in blood clotting, melanization and inducing production of antimicrobial peptides/proteins, inhibition of hyphal growth, even digesting the invading fungus in the host heamolymph. At the same time, Imp-7 in the infected larvae may mediate importing relevant proteins into the nucleus and promote cell differentiation of the host. Furthermore, we identified some *B. bassiana* genes in the infected silkworm by analysis of differentially expressed genes during different time points of infection. They might be important for *B. bassiana* in infecting the silkworm. More *B. bassiana* genes had been detected at 15 hpi and 24 hpi than at 8 hpi, suggesting that only a part of the *B. bassiana* genes were required in the very early stage of infection in fighting with the silkworm immune system. The results of this study provided a new overview of the host response to fungal infection and insights for further investigation of the complex interactions between *B. bassiana* and the silkworm. Subsequent investigations should include the functional assessment of individual DEGs that may be involved in direct or indirect anti-fungal activities.

## Materials and Methods

### Silkworm Strain

The silkworm strain, Dazao, provided by the Sericultural Research Institute of Chinese Academy of Agricultural Sciences, was used in the study. The larvae were reared on fresh mulberry leaves at 25°C. The newly exuviated larvae of the third instar were used for the experiments.

### Treatment with *B. bassiana* Conidia

Conidia of *B. bassiana* were diluted to a concentration of 4×10^6^ spores/ml with sterile distilled water. The larvae were immersed in the conidia solution for 10 s. The control ones were immersed in the sterile distilled water for the same period. Then all the larvae were reared at the higher temperature and humidity of 28°C and 95% RH.

### Collection of Samples

Generally, the *B. bassiana* spores adhered to the body of the silkworm larvae will germinate at approximately 6-8 h post-inoculation (pi). Therefore, the larvae were collected at 8 hpi, 15 hpi and 24 hpi respectively and numbered correspondingly as ZT1/ZC1, ZT2/ZC2 and ZT3/ZC3 (ZT was the tested samples and ZC was the control ones) in the following sections. The samples were immediately frozen in liquid nitrogen and stored in −80°C. Some inoculated and control larvae were reared until the inoculated ones were dead to confirm the infection by *B. bassiana*.

### Isolation of Total RNA

The total RNA was extracted from the whole larvae of both the *B. bassiana*-infected and the control by using Trizol reagent (Invitrogen). mRNA was purified by Oligo(dT) magnetic beads, then used to synthesize the first and second-strand cDNA.

### Construction of Digital Gene Expression Profiling

The 5′ sticky ends of the cDNA fragments were generated by endonuclease *NlaIII*, which recognizes and cuts off the CATG sites. Then the cDNA fragments are purified by magnetic beads and the Illumina adaptor 1 is ligated to the 5′ end of the cDNA fragments. Another endonuclease, *MmeI*, is used to digest the cDNA fragments, cutting at 17 bp downstream of the CATG site to produce the tags with adaptor 1. After removing 3′ fragments with magnetic beads precipitation, Illumina adaptor 2 is ligated to the 3′ ends of tags, then tags with different adaptors are acquired and a 21 bp tag library is constructed. After 15 cycles of linear PCR amplification, 105 bp fragments are obtained and purified by 6% TBE PAGE Gel electrophoresis. After denaturation, the single-stranded molecules are entered and fixed onto the Illumina Sequencing Chip (flowcell). Then four types of nucleotides which are labeled by four colors are added in and sequencing with the method of sequencing by synthesis is performed. Each tunnel will generate millions of raw reads with 49 bp length.

### Data Analysis of DGE Profiling

#### Clean Tags

The sequencing-received raw image data were transformed into raw sequences data by base calling. Raw sequences data have 3′ adaptor fragments, a few low-quality sequences and several types of impurities. Clean tags were generated by removal, from the raw sequence data, of 3′ adaptor sequence, empty reads (reads with only 3′ adaptor sequences but no tags), low quality tags (tags with unknown sequences 'N'), tags with a copy number of 1 and tags which were too long or too short, leaving only the tags of 21 nt long.

### Annotation of Gene Expression

Clean tags can be used after quality assessment, saturation analysis and experimental repeatability analysis. Quantity assessment includes the classified statistic of total and distinct tags. The sequencing saturation analysis was used to estimate whether the sequencing depth was sufficient for the transcripts coverage or not. When sequencing amount (total tag number) reaches 2 M or higher, the number of detected genes almost ceases to increase. Experimental repeatability analysis refers the correlation analysis of two parallel experiments. The closer correlation value approaches to 1, the better the experimental repeatability is.

After quality assessment, saturation analysis and experimental repeatability analysis, all sites of CATG were searched against the reference database (the silkworm database, ftp://silkdb.org), a virtual library containing all the possible CATG + 17 nt length sequences was constructed. All the clean tags were mapped to the reference sequences and only 1 bp mismatch is considered. Clean tags mapped to multiple genes are filtered. Remainder clean tags are designated as unambiguous clean tags. The number of unambiguous clean tags for each gene is calculated and normalized to transcripts per million clean tags (TPM), then the normalized gene expression level are obtained. As the extracted RNA and sequencing librares may contain *B. bassiana* genes, we also mapped unambiguous tags to the *B. bassiana* genome [58] to examine the expression of *B. bassiana* genes with same procedures.

### Differential Expression Detection of Genes

After obtaining the normalized gene expression level, the differential expression (DE) of each gene across samples was compared. According to Audic's description, DE detection of genes or tags across the samples was performed [100]. The genes with False Discovery Rate (FDR) ≤0.001 and the absolute value of log_2_ Ratio ≥1 were defined as the differentially expressed genes (DEGs).

### Analysis of Gene Ontology and KEGG Pathway

The gene ontology (GO) (http://www.geneontology.org/) was used to predict the possible functions of all differentially expressed genes. Pathway analysis were performed using Molecular Homological Description System 2.0 (MAS, 2.0, http://www.capitalbio.com) developed by CapitalBio Corporation. Based on KEGG, pathways with Q-value ≤0.05 were defined as the significantly enriched pathways. P value was calculated by the relative transcript level of gene and Bonferoni corrected.

### Validation by Real-time qPCR

To validate DEGs in the libraries, 22 DEGs were selected for real-time qPCR confirmation. 12 primer sequences and related information are shown in table 4.

**Table 4:** Primers used in real-time qPCR for validation of DEGs.

Primer number	genes	primers
SP1	neutral lipase	TTGGCTCTGCGTTCTGGGTTAT
		CCTCGGTACGTTTGTGGATTTG
SP2	Bm8 interacting protein 2d-4 precursor	CCTGTCTCGGTAACACTCAATGCG
		TAGTAGCGGGTCGAGGCCACTACT
SP3	glucose transporte.	TAAAATTCGAGGCAGCAGCGAT
		GGCGTCTTCTTCAGACAAACCG
SP4	hypothetical protein KGM_13211	AAGGTAATGGCTACATTCCAACATCG
		ATTTCGGCTATCATCTCGTTCAACTG
SP5	troponin C	AAGGTAATGGCTACATTCCAACATCG
		ATTTCGGCTATCATCTCGTTCAACTG
SP6	ecdysone-induced protein 63F 1	GACGACCTCATCTCCGATCTCATT
		GCTTGTATTTTTGTCACCCACTGC
SP7	amidase	CCGTAATGGCGACGAAGAATCAA
		GCCCTAACAGAACTCACGCACAC
SP8	vacuolar ATP synthase subunit D	GATGGTTCTGATACCTACGAGTTGGC
		GCTTTACAGCGCTCTGGAAGTTCTT
SP9	serine protease 13	TCAGTATGCGGCTCATCTATGCTCAG
		ACGAAGACAGTGGTAGTTCCAAAGGC
SP10	putative protein phosphatase-5	AGAAATCACTGCACCAAGCACCCAA
		GGAGGCATCACCTAAAGCATAACCG
SP11	hemolymph proteinase 18	GCGTGCTTGTATTCTGGTG
		GTTCGAAGGGTAGCGTGTC
SP12	peptidoglycan recognition protein S6 precursor	AATTCCTTAGGCTGGGGTGA
		CCGTGGACAGAAGCTTTTTT
	β-actin	AATGGCTCCGGTATGTGC
		TTGCTCTGTGCCTCGTCT

According to the SYBR Premix Ex Taq™ Kit (TaKaRa) protocol, the reactions were run on an Opticon lightcycler (BioRad) using 20 µL reaction system. Reaction procedures were: 95°C 5 s, 45 cycle at 60°C 10 s, 72°C 10 s. All samples were done in triplicate. The Ct values obtained from β-actin (a housekeeping gene of silkworm) amplification in the same plate were used to normalize the relative expression levels. The data of relative expression levels were analyzed and normalized relative to β-actin transcript levels by an Opticon monitor analysis software (MJ Research). The mean value ± SD was used for analysis of relative transcript levels for each time point using the 2^−ΔΔct^ method [59].

## Supporting Information

Figure S1 **Saturation evaluation of different expression in each library.**
(DOC)

Table S1 **Distribution of antisense and sense transcripts in DGE libraries.**
(XLS)

Table S2 **DEGs with GO annotation identified at 8 hpi.**
(XLS)

Table S3 **DEGs with GO Annotation identified at 15 hpi.**
(XLS)

Table S4 **DEGs with GO annotation identified at 24 hpi.**
(XLS)

Table S5 **List of detected fungal genes between 8 and 15 hpi.**
(XLSX)

Table S6 **List of detected fungal genes between 8 and 24 hpi.**
(XLSX)

Table S7 **List of detected fungal genes between 15 and 24 hpi.**
(XLSX)
