# Supplementary material for: Transcriptome Analysis of Silkworm, Bombyx mori, during Early Response to Beauveria bassiana Challenges
Source: PLoS One. 2014 Mar 11;9(3):e91189. doi: 10.1371/journal.pone.0091189 (PMC3949756; doi:10.1371/journal.pone.0091189)
Supplement: Figure S1 — Saturation evaluation of different expression in each library. (DOC) [file pone.0091189.s001.doc]

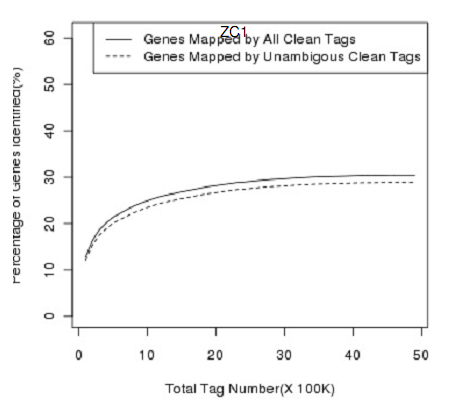

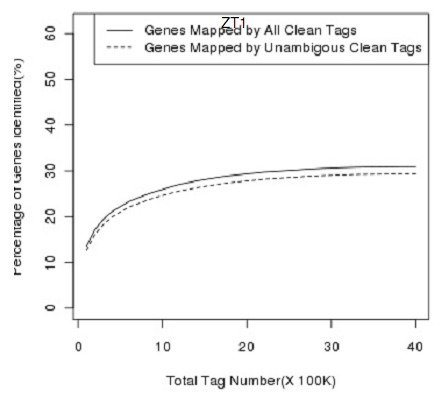

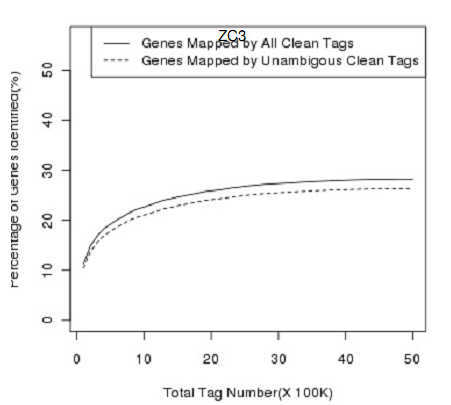

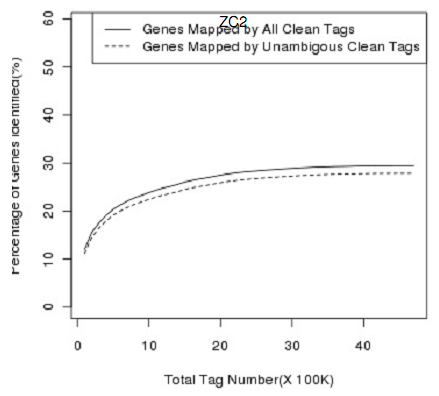

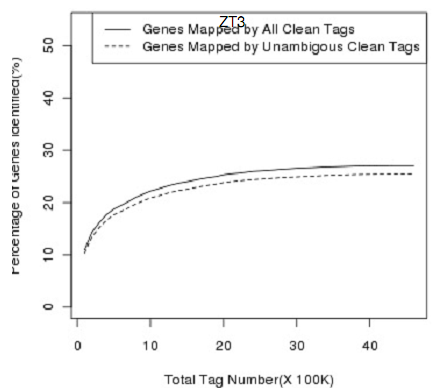

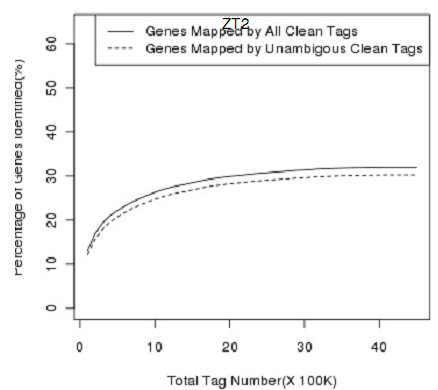


Fig S1 Saturation evaluation of different expression in each library

ZT1, library of infected larvae at 8 hpi; ZC1, library of control larvae at 8 hpi; ZT2, library of infected larvae at 15 hpi; ZC2, library of control larvae at 15 hpi; ZT3, library of infected larvae at 24 hpi; ZC3, library of control larvae at 24 hpi;
